# Health information needs regarding diabetes mellitus in China: an internet-based analysis

**DOI:** 10.1186/s12889-020-09132-3

**Published:** 2020-06-23

**Authors:** Tian-Hao Wang, Xiao-Feng Zhou, Yuan Ni, Zhi-Gang Pan

**Affiliations:** 1 Department of General Practice, Zhongshan Hospital of Fudan University, Shanghai, 200032 China; 2 Ping An Healthcare and Technology Co., Ltd., Shanghai, 200001 China

**Keywords:** Machine learning, Diabetes mellitus, Question classification, Consumer health information, Health problem

## Abstract

**Background:**

Today,. most people use the Internet to seek online health-related information from general public health-related websites and discussion groups. However, there are no Internet-based analyses of health information needs pertaining to diabetes in China until now. With the development of artificial intelligence,we can analyzed these online health-related information and provide references for health providers to improve their health service.

**Methods:**

We have done a study of statistically analyzing the questions about diabetes collected from 39 health website, the number of which is 151,589. We have divided these questions into 9 categories using a convolutional neural network.

**Results:**

The diabetes problems of consumer are presented as follows, diagnosis: 34.95%, treatment: 25.17%, lifestyle: 21.09%, complication: 8.00%, maternity-related:5.00%, prognosis: 2.59%, health provider choosing: 1.40%, prevention: 1.23%, others: 0.58%, The elderly are more concerned about the treatment and complications of diabetes, while the young are more concerned about the maternity-related and prognosis of diabetes. The diabetes drugs most frequently mentioned by consumers are insulin, metformin and Xiaoke pills, The most concerned complication is caidiovascular disease and diabetic eye disease.

**Conclusion:**

Diabetes health education should focus on how to prevent diabetes and the contents of health education should be different for differernt age groups;on diabetes treatment, the use of insulin and oral hypoglycemic drugs education should be strengthened.

## Background

Diabetes mellitus is a metabolic disorder characterized by excessive glucose levels and less insulin or absent of insulin hormone in the blood circulation [1]. As one of the world’s most prevalent diseases, diabetes is an expanding global health problem [2]. The number of cases with diabetes has more than quadrupled during the past 3 decades in the world, rising from 108 million in 1980 to 422 million in 2014, and the global prevalence of diabetes has risen from 4.7% in 1980 to 8.5% in 2014 among adults over 18 years of age [3]. In addition, the number of people with diabetes is going to be projected to 642 million across the world by 2040 [4], which predominantly occur in low and middle-income countries [5]. As a major cause of blindness, kidney failure, heart attacks, stroke and lower limb amputation, diabetes was estimated to cause 1.6 million direct deaths in 2016, and identified by WHO as the seventh leading cause of death in 2016 [6].

China is the most affected country by diabetes over the world, and currently, more than 114 million people are estimated to have diabetes in China [7]. During the past 30 years, the prevalence of diabetes has shown a dramatic rise in China, with less than 1% in 1980, 5.5% in 2001, 9.7% in 2008, and 10.9% in 2013 [8–12]. Therefore, diabetes is widely accepted as a big challenge for China in the twenty-first century, which has the greatest prevalence as well as the largest absolute disease burden of diabetes in the world [13].

Since its advent, both the number of Internet users and Internet access rate has continued to increase across the world during the past 3 decades (https://www.internetworldstats.com/stats.htm). Currently, the Internet is a major information tool, both in people’s professional and private lives. and the advent of Internet has given rise to the exponential growth of information resources which has invariably provided a wider means of access to professional in meeting their immediate information needs [14]. Among the Internet users, most use the Internet to seek online health-related information from general public health-related websites and discussion groups [15].

According to the China Internet Network Information Center, the number of Internet users in China reached 802 million in 2018, and the Internet penetration rate was 57.7% [16]. The Internet has become an important carrier for consumers to express their health information needs and search for health information. Furthermore, patients with chronic diseases are more willing to share experiences and seek help via the Internet [15]. In order to meet the needs of the market, there are a large number of websites, apps and discussion groups created in China that start offering online disease and health counseling services for consumers [17]. As a result, these sites have accumulated a large number of disease-related questions, from which we can mine a lot of valuable information about what the patients are concerned, and health providers like hospitals can make use of these questions raised by patients to improve their services such as patient health education, patient follow-up,et al.. However, there are no Internet-based analyses of health information needs pertaining to diabetes in China until now. Hereby, we report the health information needs regarding diabetes mellitus in China based on “39 Health” (http://www.39.net/), a leading website for Chinese people seeking disease and health information.

In some previous study, Haihong Guo et el. analyzed hypertension-related questions, which studied a very limited amount of data of 2000 questions [18]. Zongcheng Jia et el. studied cancer-related questions with a data of 1000 questions [19]. As far as we are concerned, there is occasionality in the distribution of such a small amount of data, which in general can not represent the reality. And with the development of artificial intelligence, some traditional machine learning algorithm like SVMs and Naive Bayesian have been used to classify questions by feeding some features as BOW or TF-IDF extracted from the dataset into the algorithm [20], which is not using the state of art technology.

To analyze the diabetes-related questions, we used a convolutional neural network to classify these questions, which is capable of capturing semantic level information. Based on the results of the classifier, we have further explored the hidden information in the data.

## Methods

### Data collection

In order to reflect the real distribution of the diabetes-related questions as much as possible, we designed a spider specific for a Chinese health-seeking website (http://www.39.net/) to capture all diabetes-related questions without any selection or filter, of which the total number was 151,589. Since these questions are all raised by patients, most of which don’t have a medical background, these questions about diabetes are very colloquial and there are a lot of misspellings. For instance, many patients may type “二甲双胍” (metformin) as “二甲双瓜” (metformin). And some consumers may merge several questions into one single question like “Do I have diabetes? Is it necessary to take medicines? Which treatment is better?” The diversity and complexity of dataset is also a huge challenge for us, which proposes higher requirements for the robustness of our model.

### Classification of diabetes-related questions

According to previous classification protocol [18–21], together with the specific characteristics of diabetes, all diabetes-related questions are classified into 9 categories, including diagnosis, treatment, lifestyle, complication, maternity-related problem, prognosis, health provider selection, prevention and others. The “others” category represents some description irrelevant to disease. Using such a schema, each question can be classified into one of the classifiers.

### Word2vec

Word2vec is an efficient model for learning high-quality distributed vector representations that capture a large number of precise syntactic and semantic word relationships [22]. Unlike traditional bag of words representations, words are projected from a sparse, 1-of-V encoding using one-hot encoding (here V is the vocabulary size) onto a lower and dense dimensional vector space via a hidden layer. In general, the dimensions of vectors are set between 100 and 500. In such dense representations, semantically close words are likewise close in Euclidean or Cosine Distance in the lower dimensional vector space.

Since Word2vec uses unsupervised learning method, it’s very efficient and easy to use. It has been shown that performance can be improved by training Word2vec model using domain-specific data before training a downstream neural network [23]. To train our own word2vec model, we have fed a large corpus of text into the model, including all the diabetes-related questions and a lot of medical-related documents. In our model, the dimension of the vector is set at 300. As a result, each word can be represented in a distributed vector, containing syntactic and semantic information. Therefore, we can calculate the similarity that can be represented by the cosine similarity between the 2 vectors correspondent (Table 1). As shown in Table 1, the semantic information is well captured by the model trained. Such a well-trained model can improve the performance of our neural network model.

**Table 1:** Word similarity

Word1	Word2	Similarity
diabetes	hypertension	0.62
diabetes	apple	0.18
diabetes	fruit	0.17
diabetes	vegetable	0.12
fruit	hypertension	0.14
fruit	apple	0.63
fruit	vegetable	0.87

### Convolutional neural network (CNN)

There are two main deep neural network architectures in natural language processing, including CNN and recurrent neural network (RNN). CNN is mainly used for classification tasks like sentiment classification since sentiment is usually determined by some key phrases; RNNs is mainly used for a sequence modeling task like language modeling as it requires flexible modeling of context dependencies [24]. Since our task is to classify questions, CNN is chosen as our basic network architecture.

**Fig. 1:**
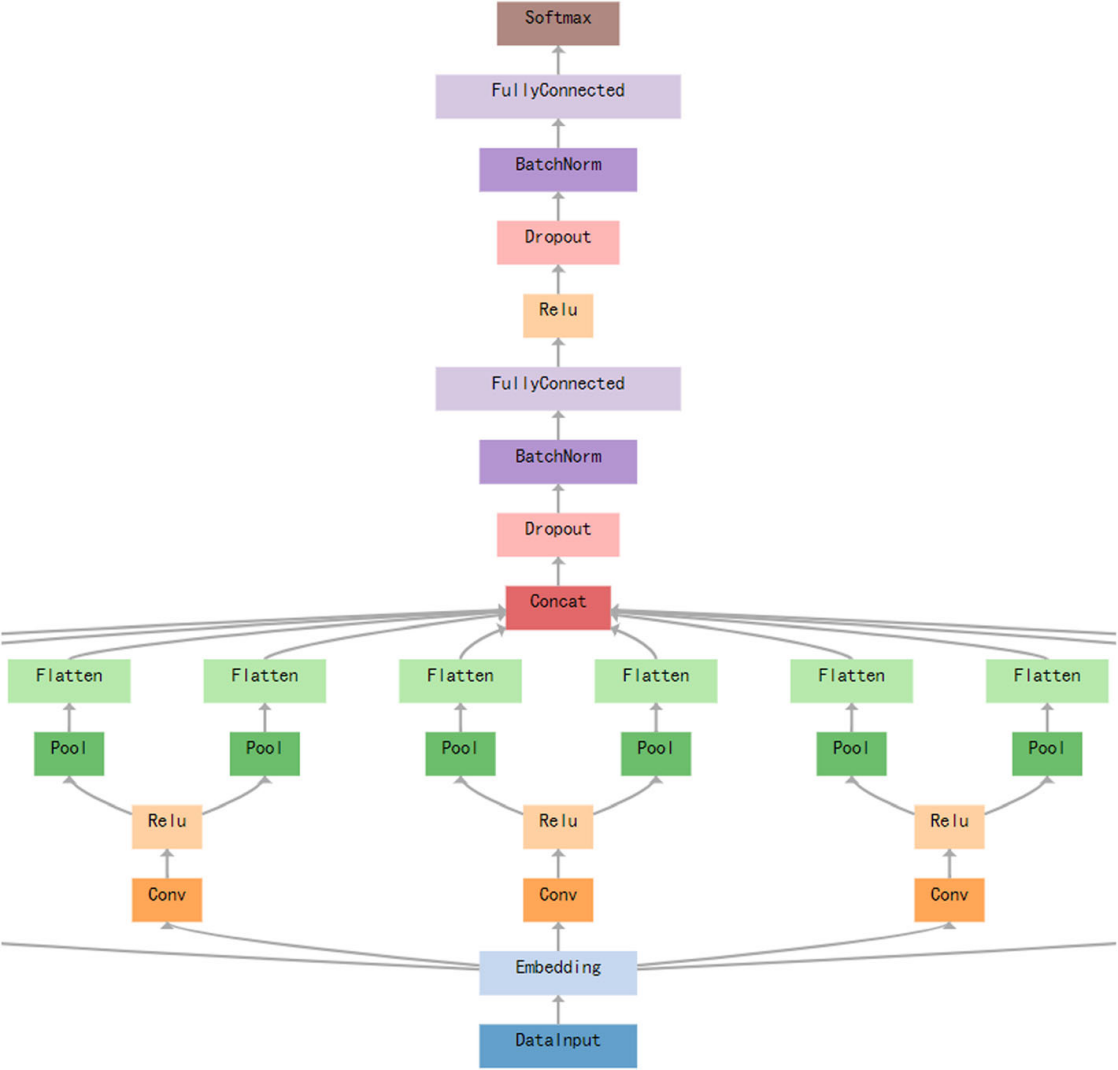
Main model architecture

With the help of the Word2vec model, sentences can be embedded into a matrix. After that, sentences can be treated just as an image which is originally represented by a matrix. In this study, we used a multi-channel CNN which is largely used in our other classification tasks, and have also been proven to achieve excellent results. The main model architecture is shown in Fig. 1.

### Annotation and training

To train a deep neural network, it’s essential to feed labeled data into the deep model. We manually annotated 1000 questions randomly selected from the dataset in the first place. Using such a small amount of data, we have been able to train a model that performs not very well but can already identify certain patterns according to the limited training data. Then, we use CNN to predict these unlabeled data and use the results as references to help us speed up the annotation process. The output of the model represents the probabilities that one sentence is classified into each category. Those sentences with low probabilities mean that the model fails to identify the patterns in the questions, which in most cases, represents that there is no similar pattern in the labeled data. Therefore, we need to annotate more data with new patterns for the model to learn. In this study, we defined the non-captured pattern threshold at 0.7, and the captured pattern threshold at 0.95, indicating that if the prediction probability of a question was below 0.7, we judged that the pattern of this sentence cannot be recognized by the model; in contrast, the prediction probability of a question that was above 0.95 suggested that the sentence was well recognized. Next, we manually annotated another 500 questions randomly selected from the unrecognized questions.

By repeating the training and annotation process above 4 times, we were able to train a well performing CNN.

### Statistical analysis

All data were managed using Microsoft Excel 2010 (Microsoft; Redmond, WA, USA), and all statistical analyses were performed using the statistical software SPSS version 17.0 (SPSS, Inc.; Chicago, IL, USA). Descriptive analysis and chi-square analysis were used to compare the percentages between groups, with a *P* value < 0.05 considered statistically significant.

## Results

### CNN performance

We have annotated 3000 questions in total by means of the annotation and training strategy mentioned in the method section. All labeled data were divided into 2 sets, the training set occupying 80% for training our model and the test set occupying 20% for evaluating our model. The accuracy of our neural network was 96.7% in the test set.

Using such an annotation and training strategy, we can avoid annotating the same patterns all the time and be able to feed more new patterns to our neural network, which can largely improve the performance and robustness. Here is an example of the classification result to prove the robustness of our deep neural network:

- Question without misspelling:

长期吃二甲双胍对健康都有些什么副作用? (What are the side effects of long-term consumption of metformin on health?)

- Question with misspelling:

长期服用二甲双瓜有什么副作用? (What are the side effects of taking metformin for a long time?)

Both questions are properly classified into the same class: treatment. The classifier works fine even if there is a spelling mistake in the sentence.

### General features of diabetes-related questions captured

**Fig. 2:**
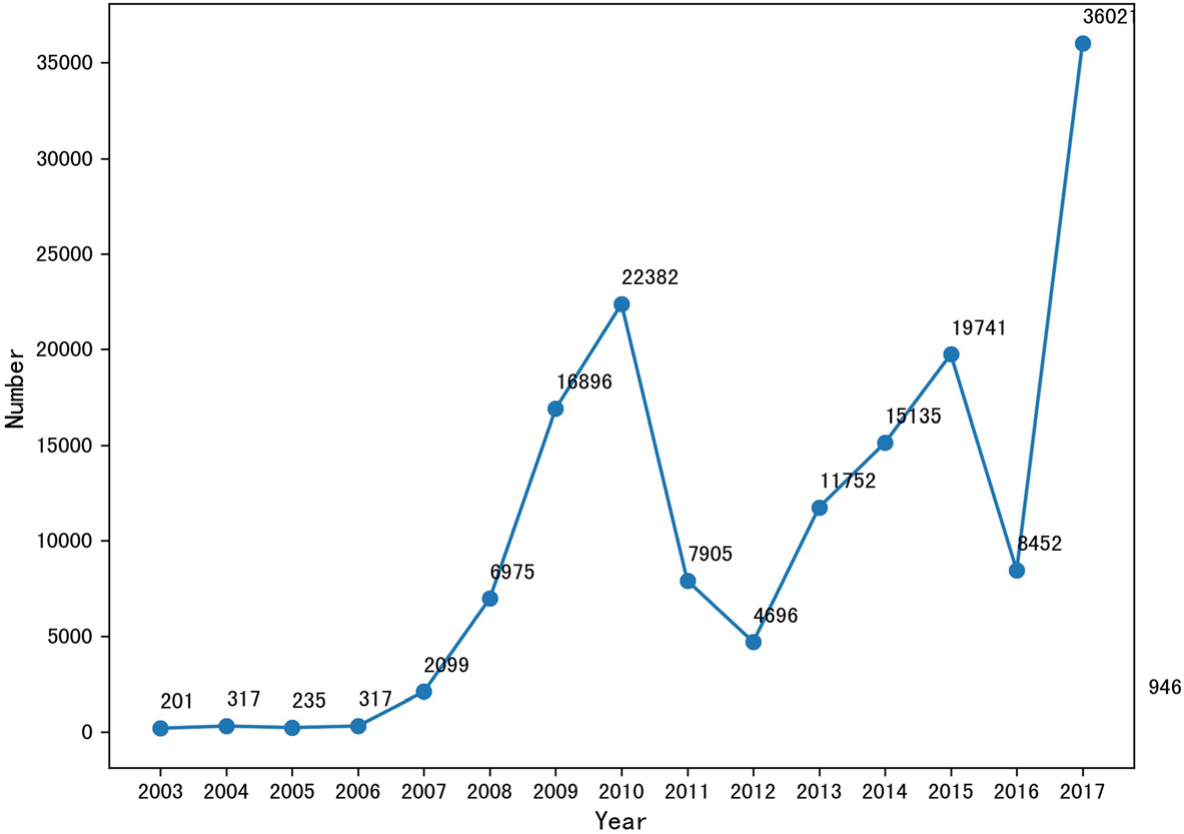
Trends in number of problems

Totally, there were 151,589 questions about diabetes put forward by 39 healthy online patients during the period from 2003 to 2017, and the number of questions appeared a gradual rise over the study period on the whole (Fig. 2). In terms of the questions, women were more willing to seek online health information than men (61.3% vs. 38.7%).

### Classification of diabetes-related questions

**Fig. 3:**
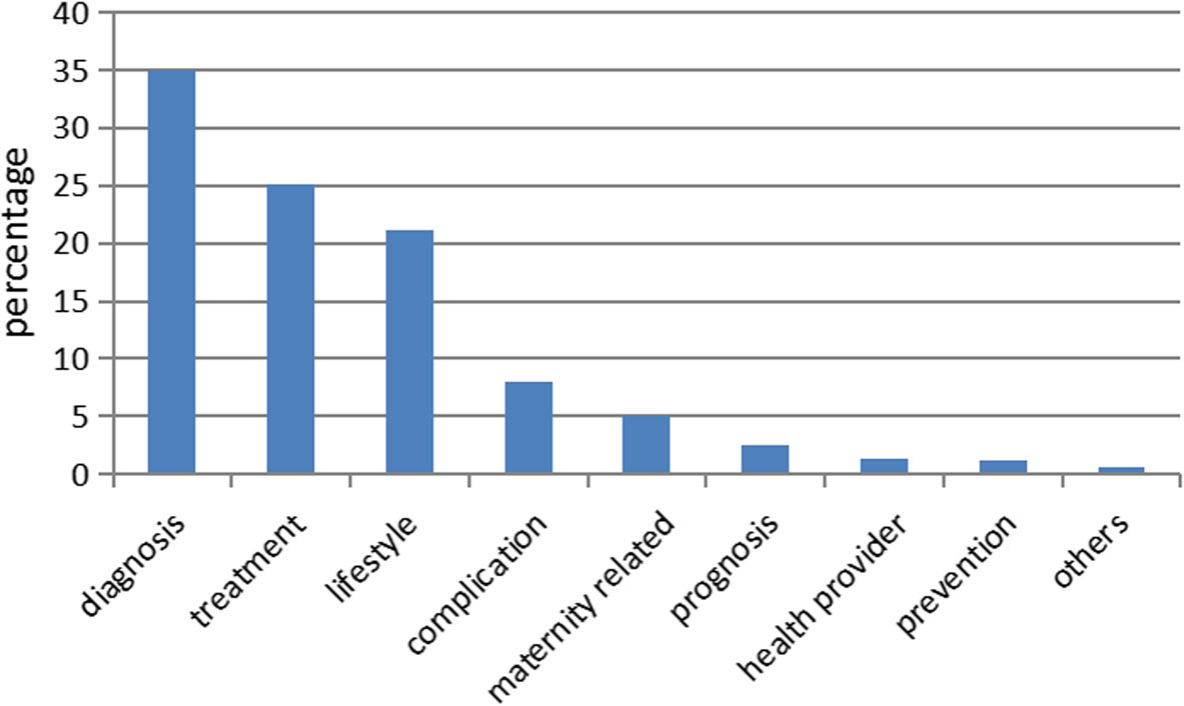
Classification of diabetes-related questions

The classification result of the number of 151,189 question is presented in Fig. 3,over 50% questions were concerned about diabetes diagnosis (34.95%) and treatment (25.14%), while little attention (1.23%) was paid to diabetes prevention. These data suggested that Chinese populations care more about diabetes after the development of diabetes.

There was no gender-specific significant difference in the classification of diabetes-related questions (*χ*^2^ = 2.033, *P* = 0.98), and the concerns were significantly difference between consumers aged 20 to 40 years and 60 years and older (*χ*^2^ = 33.528, *P* = 0.006). Young and middle-age consumers (20 to 40 years) were more concerned about maternity-related issues (16.17% vs. 0.25%) and prognosis (3.54% vs. 1.99%), while older populations (60 years and older) were more concerned about diabetes treatment (32.12% vs. 16.86%) and complications (10.05% vs. 6.31%) (Table 2).

**Table 2:** Classifcation result of different age and different gender

Group	Classification									***P*** value
	Diagnosis	Lifestyle	Treatment	Maternityrelated	Complication	Health provider choosing	Prognosis	Prevention	Others	
Age										
20-40 yrs	29.93	24.02	16.86	16.17	6.31	1.16	3.54	1.58	0.43	*X*^2^ = 33.670 *P* = 0.006
40-60 yrs.	31.95	26.13	25.07	1.37	8.76	1.52	3.15	1.56	0.48	
> 60 yrs	30.85	21.96	32.12	0.25	10.05	1.46	1.99	0.74	0.57	
Gender										
Male	33.78	23.30	25.20	2.77	8.64	1.42	3.08	1.27	0.58	*X*^2^ = 2.088, *P* = 0.98
Female	35.78	20.21	24.60	6.57	7.35	1.35	2.36	1.23	0.52	

**Fig. 4:**
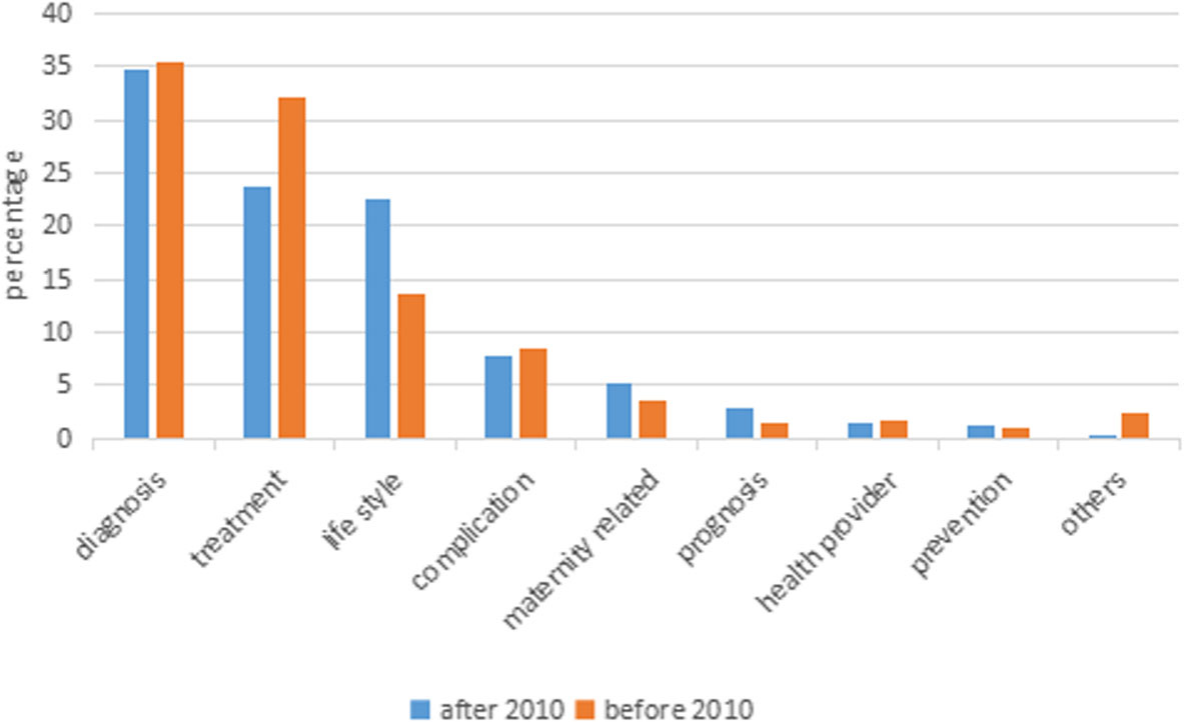
Comparison of classification before and after 2010

The proportion of consumers’ care about lifestyle approximately doubled after 2010 relative to before 2010 (22.59% vs. 13.71%; χ^2^ = 2.654, *P* = 0.103), which may be attributed to the increase in the awareness of the critical role of lifestyle in the management of diabetes with the socioeconomic development and the improvements in human health awareness (as shown in Fig. 4).

### Drugs and complications

About the medications used for treatment of diabetes, consumers raised more questions about insulin, metformin, gliclazide, and xiaokepill. Since 2007, the care about Xiaokepill decreased gradually, while the use of acarbose and glimepiride appeared an increase. But new drugs such as acarbose, siglitin, glimepiride and pioglitazone are still few. And the most concerned problems about metformin are the indications and dosage of medicines.

The most four frequently mentioned complications included diabetic cardiovascular disease and diabetic eye disease (including retinopathy and cataract), diabetic nephropathy, diabetic foot and diabetic cardiovascular disease. Further subgroup analysis showed that consumers were concerned about diagnosis (44.05%), treatment (40.66%) lifestyle (10.19%), prevention (2.95%) and prognosis of diabetic complications (2.15%).

About classification of diabetic complications, there are significant differences in concerns among different age groups (× 2 = 452.31, *P* = 0.000). Young people pay more attention to diabetic nephropathy, while older people over 60 pay more attention to diabetic cardiovascular disease (Table 3).

**Table 3:** Complications of different age and different gender

	Cardiocascular disease	Nephropathy	Diabetic foot	Diabetic eye disease	Ketosis	Neuropathy	Cerebrovascular disease	***P*** value
Age								
20-40 yrs	999	338	241	217	106	76	65	X^2^ = 452.31 *P* = 0.000
40-60 yrs	2661	660	563	776	138	149	185	
> 60 yrs	2741	268	224	586	91	67	178	

## Discussion

According to the latest epidemiological survey, the prevalence of adult diabetes in China has reached 10.9% in 2013, and the prevalence of pre-diabetes has also reached 35.7%,and the prevalence of diabetes in adults younger than 40 years old was 5.9%,However, the treatment rate of diabetes in China is only 32.2%, and the control rate of diabetes is only 49.2% [10]. Because diabetes is a long-term chronic disease, the daily behavior and self-management ability of patients is one of the keys to the control of diabetes, The patients who receive self-management education of diabetes had better control of blood sugar than those without education. At the same time, they had more positive attitude, scientific knowledge of diabetes and better self-management behavior of diabetes [25]. Therefore, health education for diabetic patients or high-risk groups of diabetes should be strengthened in the future.

With the development of information technology, more and more patients with diabetics and their families are willing to get health information through the Internet to confirm the diagnosis of disease, patients’ physical condition, to share experiences in treatment, diet control, and to obtain social emotional support, so as to learn self-management of diabetes [14]. According to the Europe digital health reports published in 2014, 75% of Europeans believe that the Internet is a good resource for seeking health information [15]. Wagner and colleagues [26] found that 52% of patients with diabetic would look for health informationthrough the Internet. Our study shows that the number of online questions about diabetes mellitus from 2003 to 2017 is on the rise except 2010 to 2012 and 2015 to 2016, In 2017, the number of online questions about diabetes mellitus was more than 10 times that of 2007. This shows that domestic consumers are increasingly searching for health information about diabetes through the Internet, and female consumers are more willing to ask questions online than male consumers.

Many studies have also shown that online health-related knowledge can influence individual health decisions and behaviors [27]. on one hand,To study the diabetes health problems of consumers can let medical service providers understand their needs and provide better health care services;On the other hand, public information on diabetes health problems is largely influenced by medical service providers, such as treatment, drugs, diet and exercise. In order to understand these problems, consumers often ask for help in the internet, therefore, Understanding the needs of patients who have diabetics and what kind of problems they are anxious about are crucial for doctors, hospitals and other health care providers, which can be used to ameliorate patient education service and help patients to improve their disease management skills.

The diabetes problems on the internet were classfied into 12 topics by Zhang and Zhao [28]:etiology and pathophysiology, signs and symptoms, diagnosis and examination, organs and body parts, complications and related diseases, drug treatment, treatment, health education and information resources, prognosis, society and culture, lifestyle and nutrition. Nutrition, diagnosis and examination Symptoms and related diseases are the three topics most frequently mentioned by consumers. Foxand Duggan [29] and others found that the most topics for diabetic searched by patients were weight loss or weight control. This article divides the diabetes problem into nine categories:diagnosis, treatment, lifestyle, complication, maternity related, prognosis, health provider choosing, prevention, others. The analysis results show that domestic consumers are mainly concerned about the diagnosis of diabetes, treatment, lifestyle, complication, maternity related, Although this study suggests that all consumers pay more and more attention to diabetes lifestyle, only 1.23% of the questions about diabetes prevention, which indicates that The purpose of most consumers is not to know how to prevent diabetes, but to confirm the diagnosis to others after finding diabetic signals or to consult diabetics about their current treatment.

There is no difference in the concerns of different genders, but the concerns of different age groups are different.. Young people are more concerned about maternity-related issues, who are at an age suitable for childbearing. They may worry about problems like whether diabetes will be passed on to children or whether people with diabetes have fertility. While older people are more concerned about treatment and complications to alleviate the impact of diabetes on life. Many people in this age are using oral medication or insulin therapy. They are more eager to know the drug usage, efficacy and adverse reactions.

As the treatment of diabetes, insulin, metformin, Xiaoke pill and gliclazide were the most frequently asked drugs. Insulin plays important roles in controlling hyperglycemia. Compared with oral medicine, insulin therapy involves more links, such as drug selection, treatment plan, injection device, injection technology, SMBG, action based on blood sugar monitoring results, etc. [25, 30] Patients who begin to use insulin should master self-management skills related to insulin therapy through targeted education, understand the risk factors, symptoms and self-rescue measures of hypoglycemia,. Therefore, diabetic patients and their families have more problems in this regard,which suggests that health providers should teach patients and relevant medical staff more knowledge of insulin, so as to improve their attitude towards diabetes.

In terms of oral hypoglycemic drugs, metformin is the most concerned drug,which shows that the status of metformin in treatment is increasingly recognized, and metformin should be recommended as the first choice for diabetes treatment in chinese diabetes gudline. Xiaoke Pill is a kind of Chinese patent medicine, which is favored by many consumers because of the cheap price. Xiaoke Pill has the same hypoglycemic effect as glibenclamide, Compared with glibenclamide, Xiaoke Pill has a lower risk of hypoglycemia and a more significant effect on improving TCM symptoms related to diabetes mellitus [31]. As for other oral medicines, with the increase of years, more and more new hypoglycemic drugs have been used in clinical practice, but the public’s attention to new drugs has not increased significantly, suggesting that doctors should be more active in the application of new diabetic drugs.

In terms of diabetic complications, consumers pay more attention to diabetic eye diseases, followed by diabetic nephropathy and diabetic foot. This is not consistent with that the common complications of diabetes in hospitalsare diabetic cardiovascular disease, diabetic nephropathy and diabetic foot [32]. It may be related to the way we collect data (for example, some questions about diabetic cardiovascular complications may be classified into the heart Department. In the classification of complications, consumers pay more attention to the diagnosis and treatment of complications, but pay little attention to how to prevent complications, suggesting that medical professionals should give more guidance to patients in the prevention of diabetic complications.

The advantage of this study is that the data of health problems are all collected from 39 Health websites, which span a wide range of time and are collected without filters. It may represent the true distribution of diabetes-related problems among consumers to some extent. However, there are also some shortcomings in this study: first, There is no relevant analysis of the geographical and economic situation of the online questioners,second,the problems related to sports are not counted; last, there is no further detailed analysis of drug treatment problem of consumer concerns such as side effects, usage, adverse reactions, etc. It needs to be supplemented and improved in future studies.

## Conclusion

Diabetes health education should focus on how to prevent diabetes and the contents of health education should be different for differernt age groups;on diabetes treatment, the use of insulin and oral hypoglycemic drugs education should be strengthened.

## Data Availability

The datasets used and/or analysed during the current study are available from the corresponding author on reasonable request.

## Abbreviations

SVM: support vector machine
BOW: bag of words
TF-IDF: term frequency-inverse document frequency
CNN: convolutional neural network
RNN: recurrent neural network
SMBG: self monitoring blood glucose
TCM: traditional chinese medicine
